# Susceptibility of different TMEM154 genotypes in three Italian sheep breeds infected by different SRLV genotypes

**DOI:** 10.1186/s13567-022-01079-0

**Published:** 2022-07-29

**Authors:** Riccardo Moretti, Stefano Sartore, Barbara Colitti, Margherita Profiti, Stefania Chessa, Sergio Rosati, Paola Sacchi

**Affiliations:** Dipartimento di Scienze Veterinarie, Università di Torino, 10095 Grugliasco, TO Italy

**Keywords:** Genotyping, infection resistance, lentivirus, ligase detection reaction, sheep

## Abstract

Small ruminant lentiviruses (SRLV) belong to the Retroviridae family and can cause various diseases. One of the most impacting diseases is visna-maedi, a complex disease characterized by long latencies and chronic progressive inflammatory events affecting the nervous system, lungs, mammary gland, and articular joints. A single nucleotide polymorphism (rs408593969, c.103G>A, missense mutation E35K) in the ovine transmembrane protein gene 154 (TMEM154) was identified as protective against small ruminant lentivirus infection in different herds worldwide. However, there is evidence in the scientific literature of a breed-specificity of this protective effect and, furthermore, there are still limited studies regarding the association between the animal genotype and the infecting virus genotype. Thus, the aim of this study was to further investigate the association between the animal genotype for the suggested protective mutation and the infecting virus genotype, in three different sheep breeds reared in northern Italy. The results obtained only partially confirmed the data available in the literature, as the protective effect was confirmed only for SRLV genotype A clusters, while other genotypes (namely B and E) infected AA and GA animals. Further studies with an experimental infection of specific virus genotypes in hosts with specific genotypes are required to confirm the larger number of cases the results obtained in this study.

## Introduction

Small ruminant lentiviruses (SRLV) are a group of worldwide distributed viruses of the Retroviridae family causing multisystemic, degenerative, and chronic diseases [1]. Among the Lentivirus genus figure caprine arthritis and encephalitis virus, visna-maedi virus, and its North American counterpart, namely ovine progressive pneumonia virus [2]. Specifically, visna-maedi (VM) is a complex, insidious production-limiting ovine lentiviral disease characterized by long latencies (both clinical and immunological) and chronic progressive inflammatory events [3]. Four different pathological manifestations of VM are described in the scientific literature and affect the nervous system (Icelandic “visna”), lungs (Icelandic “maedi”), mammary gland and articular joints [4]. From an economic point of view, VM has a negative impact on milk, reducing the output by up to 40% (estimated in the UK by Ritchie and Hosie, 2010 [5]), on wool, and on lamb production [6]. Seroprevalence of VM was moderate to high in different studies from different countries even though clinical disease was found in a reduced portion of positive animals [7]. Furthermore, the absence of a vaccine or a reliable cure contribute to the high impact of this disease on animal health and welfare [8].

As of today, monitoring programs are the only effective tool in controlling the infection, as seen in countries where VM outbreaks highly impacted sheep breeding systems (e.g., compulsory disease monitoring and eradication of the virus during an Icelandic outbreak [9]). Monitoring programs vary from culling to separating infected animals, but generally there are difficulties in applying such strategies that may therefore be unfeasible [10]. These obstacles are of particular impact when applied to local breeds, whose size and economic-related features are limiting factors. Furthermore, there are findings in the scientific literature suggesting the influence of breeds on the susceptibility to SRLV [11].

In this study, two local sheep breeds from the Piedmont region, along with a local sheep breed from the Lombardy region were investigated. Biellese (BI) is a local breed of the province of Biella in the Piedmont region (northern Italy), from which it takes its name. Formerly considered a dual-purpose breed (meat and wool), it is now reared mainly for meat production. Delle Langhe (DL) is a dairy sheep breed autochthonous of the Piedmont region (northern Italy), but native to the province of Cuneo, in the Langhe hilly area. Bergamasca (BG) is a sheep breed originating from the mountain area of the province of Bergamo in the Lombardy region (northern Italy). This breed is, however, widely reared throughout northern Italy, including the Piedmont, and it is mainly reared for meat production. According to the Italian national livestock database [12], which records all animals of a specific species and breed, the population sizes for each breed in Italy at the end of 2021 were 53 374, 5885, and 119 636 animals for BI, DL, and BG, respectively.

Given the difficulties presented before, the efforts to eradicate lentiviruses in sheep would benefit from breeding and rearing animals that are genetically resistant to SRLV infections [13] as already applied for scrapie in sheep and only prospected in goats [14, 15]. In this regard, Heaton et al. [16] identified different mutations in the ovine transmembrane protein gene 154 (TMEM154) that altered sheep susceptibility to SRLV infection. Specifically, they showed that the odds of being infected for an animal with the homozygous alternate allele A of a SNP in exon 2 (rs408593969, c.103G > A, missense mutation E35K) were 69 times smaller than the ones for animals with one copy of the wild-type allele G. Furthermore, the interaction between animal and virus genotype, in regard to the innate resistance, is still poorly investigated [17]. Given the promising findings from Heaton et al. [16], which performed their analysis on a multi-breed US sheep flock, the aim of this study was to evaluate the allelic frequencies of the E35K SNP in TMEM154 gene in the three selected local breeds and then to test the association of the different genotypes to the serological status of the same sampled animals and to the virus genotype.

## Materials and methods

### Animals and farms

A total of 155 BI sheep (146 ewes and 9 rams) were selected from three farms located in the Turin province (Piedmont, Northern Italy). Mean age of BI sheep was 2.77 ± 1.98 years (2.87 ± 1.98 and 1.22 ± 1.39 years for ewes and rams, respectively). A total of 100 DL sheep (96 ewes and 4 rams) were selected from four farms located in Asti province (Piedmont, Northern Italy). The mean age of DL sheep was 6.08 ± 4.06 years (6.24 ± 4.06 and 2.25 ± 1.89 years for ewes and rams, respectively). Furthermore, a total of 100 BG sheep (100 ewes) were selected for this study from a farm located in the Turin province (Piedmont, Northern Italy). Mean age of BG sheep was 2.60 ± 2.21 years. In all of these farms, animals were housed in barns at night, while grazing during the daytime. Blood samples were collected by jugular venipuncture by veterinarians during routine visits and stored in K3 ethylenediaminetetraacetic acid (EDTA) blood collection tubes. After transportation at 4 °C to the laboratory, each sample was split in two aliquots, one of at least 2 mL for the immunological analysis and one with the remaining blood for the genetic analysis.

### Immunologic analysis

Blood sera were analyzed for SRLV antibodies using the commercially available Eradikit™ SRLV platform (Eradikit—SRLV Starter kit, In3diagnostic, Italy), which is composed of two distinct screening and genotyping assays. Each serum was first tested in a screening assay against a mix of capsid and envelope peptides belonging to the three most divergent SRLV viral genotypes with ELISA screening plates. Then, each positive serum to the screening assay was tested in the genotyping assay in which ELISA plates are coated, on separate strips, with subunit peptides specific for genotypes A, B, and E, against which the samples are simultaneously tested for genotype-specific antibodies. Briefly, samples were diluted 1/20 in sample diluent and incubated for 1 h at 37 °C. Following three washes, a peroxidase-labeled anti sheep/goat IgG was added and the plate was incubated as described above. After a final washing step, the reaction mixture was developed with 2,2′-azino-bis-(3-ethylbenzthiazoline-6-sulfonicacid). The results were read at 405 nm and the absorbances were obtained for each serum sample against the screening antigens and the three subunits [18].

For ELISA screening, results and sample-to-positive (S/P) ratios were calculated according to the manufacturer’s instructions. Samples with a percentage of reactivity greater than 40% of positive control were considered as positive. Doubtful results (with percentage of reactivity between 30 and 40%) were considered as positive and subjected, together with positive samples, to the genotyping ELISA. For this latter, the results were calculated according to the manufacturer’s instructions and given as not conclusive (NC), positive for one (A, B, E) or indetermined (i.e., co-infection with more than one genotype).

### Genetic analysis

Genomic DNA was obtained from blood samples by extraction using the Nucleospin^®^ Blood kit (Macherey-Nagel, Düren, Germany). A region of 306 bp was amplified using the primers TMEM154 forward and reverse (TGGATGCATGGAGGGTAAGT and CTGATAGAAGCAGGAGAGAGA, respectively). The PCR was performed in a thermal cycler (Applied Biosystems^®^ 2720 Thermal Cycler) in a total volume of 25 µL, using the HotStar Taq DNA Polymerase (Qiagen, Hilden, Germany). The amplification occurred at 95 °C for 15 min, 35 cycles of 94 °C for 30 s, 50 °C for 30 s, and 72 °C for 1 min, followed by a final extension at 72 °C for 10 min. The amplified samples and controls (positive and negative) underwent 1.5% agarose gel electrophoresis, stained with ethidium bromide (Sigma-Aldrich^®^, USA), and visualized in an ultraviolet transilluminator. To further verify that the amplification product corresponded to the expected DNA fragment, 14 samples were sequenced using the SeqStudio Genetic Analyzer (Applied Biosystems by ThermoFisher Scientific, Waltham, MA, USA). The sequences obtained were then aligned to the genomic reference sequence using BioEdit software [19].

Genotyping of the samples was performed by Ligase Detection Reaction [20] using three probes designed in the amplified region. The probes were designed ad hoc and consisted of a common probe marked with a phosphate group and a fluorophore (TMEM-C, Pho-AACTGTCAGGAGACGTGCCCCCA-NED), a probe specific for the G allele (TMEM-G, TTTTTTTGTTCCCACAGGAGAGGAGGACACAG), and a probe specific for the A allele (TMEM-A, TTTTTTTCGCTCAGTTCTGTTCCCACAGGAGAGGAGGACACAA). The two allele-specific probes were designed with a size difference of 11 bp and each one had the specific SNP allele at the 3ʹ extremity. The ligase reaction was performed in a thermal cycler (Applied Biosystems^®^ 2720 Thermal Cycler) in a total volume of 10 µL using the Ampligase^®^ kit (Lucigen, Wisconsin, USA) and consisted of a denaturation at 94 °C for 2 min followed by 35 cycles of 90 °C for 30 s, and 65 °C for 2 min. Samples were subsequently analyzed by fragment analysis by capillary electrophoresis using the SeqStudio Genetic Analyzer (Applied Biosystems by ThermoFisher Scientific, Waltham, MA, USA).

### Statistical analysis

Statistical analyses and tests were performed using R software v3.6.1 [20, 21]. Post-hoc tests were performed using R package *chisq.posthoc.test* [21, 22]. Allelic and genotypic frequencies were calculated within breeds and farms using the R package *mixIndependR* [22, 23] and compared to the Hardy-Weinberg equilibrium model.

Association analyses between genotypes and resistance to the infection, along with statistical tests for the association of seropositivity with other variables, were performed only in farms according to the following criteria: animals included both resistant and susceptible genotypes; they had at least 5% of seropositivity prevalence.

Relative risk (RR) for animals to be serologically positive given a specified feature (e.g., specific genotype, breed) was calculated as [23, 24]:

$$RR = \frac{{\frac{a}{{\left( {a + b} \right)}}}}{{\frac{c}{c + d}}}$$

where: *a* is the number of positive animals with a specific feature; *b* is the number of negative animals with a specific feature; *c* is the number of positive animals without a specific feature; *d* is the number of negative animals without a specific feature.

A nominal logistic regression was fitted on data to evaluate the association between animal and virus genotype, using the R package *nnet* [24, 25]. The regression included virus genotypes as the dependent variable and animal genotypes and breed as independent variables.

## Results

Two DL farms had no seropositive animals, therefore failing the selection criteria. In addition, one BI farm had less than 5% seropositivity prevalence. Hence, animals belonging to those three farms were removed from further analyses. After pruning the dataset accordingly to the selected criteria, a total of 285 animals out of the initial 355 were further analyzed (104 BI, 100 BG and 81 DL).

The results from ELISA analysis identified a total of 135 SRLV seropositive animals (74 BI, 50 BG, and 11 DL), while 150 were seronegative (30 BI, 50 BG, and 70 DL). On the overall dataset (355 animals), an overall 27.58% of prevalence was observed, with farm specific prevalence ranging from 0 to 81.82%. In the selected farms only (i.e., after data pruning), an overall 43.73% of prevalence was observed, with farm specific prevalence ranging from 7.14 to 81.82%. A Chi-square test was performed to evaluate the relationship between seropositivity and breed, and a significant relationship was confirmed (χ^2^ = 60.97, df = 2, *p*-value < 0.001). A post-hoc test with Bonferroni correction was performed to identify the specific breed-seropositivity pairs that implied the significant relationship. Specifically, BI was statistically associated with seropositivity (*p*-value < 0.001), while DL was statistically associated with seronegativity (*p*-value < 0.001). Differently, BG was not statistically associated with neither seropositivity nor seronegativity. Furthermore, RR for seropositivity was calculated for all the breeds: BI had 1.247 times the risk of seropositivity compared to BG and 3.477 times the risk of seropositivity compared to DL, while BG had 2.788 times the risk of seropositivity compared to DL.

A Chi-square test was performed to evaluate the relationship between seropositivity and age classes, and a significant relationship was confirmed (χ^2^ = 29.47, df = 3, *p*-value < 0.001). Age classes were defined according to the findings by Leymaster et al. [25, 26], where the age threshold of 39 months (~3 years) was described. Specifically, four levels were defined, identified by the roman numerals I to IV: x < 3, 3 < x < 6, 6 < x < 9, and x > 9, respectively (where x is the age of the animal, expressed in years). A post-hoc test was performed to identify the specific age class-seropositivity pairs that implied the significant relationship. Specifically, age classes I and II were statistically associated with seronegativity (*p*-values = 0.025 and = 0.011, respectively), while age class III was statistically associated with seropositivity (*p*-value < 0.001). Differently, age class IV was statistically associated with neither seropositivity nor seronegativity. Furthermore, RR for seropositivity was calculated for all the age classes: age class I sheep had 1.775 times the risk of seropositivity compared to age class II; age class III sheep had 1.498 times the risk of seropositivity compared to age class I, 2.659 times the risk of seropositivity compared to age class II, and 1.154 times the risk of seropositivity compared to age class IV; lastly, age class IV sheep had 1.298 times the risk of seropositivity compared to age class I, and 2.305 times the risk of seropositivity compared to age class II. Age class II was protective (i.e., RR < 1) when compared to all the other age classes.

The possible effect of sex on positivity was not evaluated due to the strong imbalance in data numbers (the ratio ewes/rams were 56:1).

After checking for the amplification of the expected DNA fragment by sequencing, a Ligase Detection Reaction was performed to genotype all the animals for E35K SNP (rs408593969). The genotyping was successful for 265 out of 285 samples (with 20 samples discarded for technical issues, i.e., 7 BI, 3 DL, and 10 BG). Genotype and allele frequencies per breed are shown in Table 1.

**Table 1:** **Genotype and allele frequencies of the c.103G>A SNP for the Biellese (BI), the Delle Langhe (DL), and for Bergamasca (BG) sheep breed**.

Genotype	# BI	# DL	# BG
GG	4.12%	6.41%	0.00%
GA	52.58%	51.28%	36.67%
AA	43.30%	42.31%	63.33%

Allele	# BI	# DL	# BG
G	30.41%	32.05%	18.3%
A	69.59%	67.95 %	81.7%

Since at least one of the cells in the contingency table was less than 5, a Chi-square test was not used [26, 27]. In its place, the Fisher exact test was performed to evaluate the relationship between animal genotypes and breed, and a significant relationship was confirmed (*p*-value = 0.005). Similarly, the Fisher exact test was performed to evaluate the relationship between animal genotypes and seropositivity, not considering the different breeds. However, there was no significant relationship (*p*-value = 0.074). Therefore, the test was repeated on each different breed, and a significant relationship between animal genotype and seropositivity was confirmed only for BG and DL sheep (*p*-values = 0.001 and = 0.002, respectively). Based on these results, RR for seropositivity was calculated for all the genotypes for BG and DL sheep only. Regarding BG sheep, GA animals (EK protein isoform) had 1.548 times the risk of seropositivity compared to AA ones (KK protein isoform). Regarding DL sheep, GG animals (EE protein isoform) had 1.556 times the risk of seropositivity compared to GA sheep. Since no seropositive animals were detected among the AA sheep, RR was not calculated.

Lentivirus’ genotypes were obtained with the assay from the seropositive animals’ blood. The most represented genotype was B (53.3%), followed by A (5.9%), and E (3.7%). As expected, a small proportion of seropositive animals (7.4%) was not genotyped (O, i.e., lack of antibodies directed against the variable epitope of the capsid antigen). Lastly, indefinite genotypes (I) accounted for the 29.6% of the total genotypes. In indefinite cases, a mixed infection could not be excluded.

The subsequent analysis focused on genotyped seropositive animals, excluding animals infected by indefinite SRLV genotypes (I) or not genotyped (O), for a total of 79 animals further analyzed. Two nominal logistic regression models were fitted (i.e., with and without breed as independent variable, respectively). Breed was not statistically significant, and the AIC parameter was lower for the model without breed (85.82 and 82.55 for the models with and without breed, respectively). Therefore, the model including a breed variable was discarded. A confusion matrix was built to calculate the mismatching error made by the model when predicting the outcome variable using the training data. The mismatching error was 16.5%: while the majority of virus genotypes B were correctly assigned (96.97%), only 25% of virus genotypes A were correctly assigned, and none of the virus genotypes E were correctly assigned. *P*-values were calculated by two-tailed Z-test and were statistically significant for both B-vs-A and E-vs-A virus genotypes (0.007 and 0.006, respectively). The relative risk ratio for the A allele substitution effect in the animal genotype for being infected by virus genotype B vs virus genotype A is 10.45, while the relative risk ratio for the A allele substitution effect in animal genotype for being infected by virus genotype E vs virus genotype A is 47.42. A summary of the virus genotypes, grouped by breeds and animal genotypes are reported in Table 2.

**Table 2:** **Animal genotypes by breeds and virus genotypes (BI = Biellese, DL = Delle Langhe, and BG = Bergamasca)**.

Breed	Lentivirus’ genotype	Animals’ genotype	**#**
BI	A	GG	2
		GA	1
		AA	0
	B	GG	0
		GA	17
		AA	19
	E	GG	0
		GA	1
		AA	3
BG	A	GG	0
		GA	4
		AA	0
	B	GG	0
		GA	11
		AA	12
	E	GG	0
		GA	0
		AA	1
DL	A	GG	0
		GA	1
		AA	0
	B	GG	2
		GA	5
		AA	0
	E	GG	0
		GA	0
		AA	0

## Discussion

Since the results from Heaton et al. [15, 16], the importance of *TMEM154* gene and its E35K mutation (rs408593969) have been widely recognized. Most of the studies were performed on American, German, and Iranian herds [11, 27, 28], while few Italian data regarding the frequencies of genetic variants are available for goats only [28, 29, 30]. A first survey on Italian sheep was performed only recently by Arcangeli et al. [30, 31]. However, none of the studies available in the scientific literature considered the virus genotype.

The overall before-pruning prevalence (27.58%) was lower than other reported prevalence values (40.90%) regarding European herds, being closer to North American values (21.76% [31, 32, 32]). The effect of the age of the animal (grouped as age classes) was tested since evidence of an association was present in the literature [25, 26] and this association was confirmed (*p*-value < 0.001). Specifically, the results from this study show that younger animals (i.e., under 6 years of age) are less likely to be seropositive (*p*-values = 0.025 and = 0.011, respectively), while animals with an age comprised between 6 and 9 years are more likely to be seropositive (*p*-value < 0.001). This could be due to the increasing chances to be exposed to SRLV for each year passing.

Regarding breed-specific resistance or susceptibility to SRLV, our results are consistent with those from Molaee et al. [11]: 40% of seropositive animals in this study were homozygous for the A allele (resulting in the KK protein identified as protective by Heaton et al. [15, 16]).

Furthermore, we observed a statistically significant association (*p *< 0.001) with different breeds and seropositivity (specifically, BI had higher relative risk of being seropositivity compared to BG and DL). Similarly, there was a statistically significant (*p*-value = 0.005) relationship between breed and animal genotypes. Lastly, there was a significant relationship between animal genotypes and seropositivity only when the test was performed on each different breed (specifically in BG and DL breeds, *p*-values = 0.001 and = 0.002, respectively). These results suggest a different resistance to SRLV which is breed and genotype related, with an association between the two predicting variables. An interesting observation made is that no DL sheep with a homozygous AA genotype (KK protein isoform) were seropositive in this study, suggesting a confirmation of this genotype as protective. However, both BG and BI sheep had homozygous AA seropositive animals (KK protein isoform).

Thus, we studied the association between animal and virus genotype. We observed that no virus genotype A (which is the most common SRLV genotype in the USA) was found in AA animals (KK protein isoform) regardless of the breed, and this is consistent with the protective effect suggested by Heaton et al. [15, 16]. However, this sheep polymorphism does not have the same protective effect against other virus genotypes (namely, genotypes B and E). Regarding virus genotype B, no infections were found in GG animals (EE protein isoform) of the BI breed (no GG animals of the BG breed were available in this study). Lastly, virus genotype E was found in BI and BG breeds only, and in AA and GA animals only (KK and EK protein isoform, respectively). In addition to what has already been reported in the scientific literature, the results presented in this study could suggest different new information. First, the genotype AA of the c.103G>A SNP in TMEM154 gene is potentially protective against virus genotype A only. Second, there could be a potential susceptibility of animal carrying at least one allele A to an infection caused by virus genotypes B and E. Lastly, SRLV genotype B includes a B2 subtype which was associated to VM in sheep and is widely distributed in Italian, French, and Spanish sheep flocks, suggesting a limited impact of the SRLV genetic control program based on TMEM154 [32, 33]. Further studies possibly involving experimental infection of specific animal genotypes with specific virus genotypes, should be performed to confirm the results presented here.

## Data Availability

The datasets used and/or analyzed during the current study are available from the corresponding author on reasonable request.
